# Expression and cellular localization of cyclooxygenases and prostaglandin E synthases in the hemorrhagic brain

**DOI:** 10.1186/1742-2094-8-22

**Published:** 2011-03-08

**Authors:** Tao Wu, He Wu, Jessica Wang, Jian Wang

**Affiliations:** 1 Department of Anesthesiology/Critical Care Medicine, Johns Hopkins University, School of Medicine, Baltimore, MD, USA; 2 Department of Neurology, Changhai Hospital, Second Military Medical University, Shanghai 200433, PR China; 3 Department of Pathology, First Clinical Hospital, Harbin Medical University, Harbin 150001, PR China

## Abstract

**Background:**

Although cyclooxygenases (COX) and prostaglandin E synthases (PGES) have been implicated in ischemic stroke injury, little is known about their role in intracerebral hemorrhage (ICH)-induced brain damage. This study examines the expression and cellular localization of COX-1, COX-2, microsomal PGES-1 (mPGES-1), mPGES-2, and cytosolic PGES (cPGES) in mice that have undergone hemorrhagic brain injury.

**Methods:**

ICH was induced in C57BL/6 mice by intrastriatal injection of collagenase. Expression and cellular localization of COX-1, COX-2, mPGES-1, mPGES-2, and cPGES were examined by immunofluorescence staining.

**Results:**

In the hemorrhagic brain, COX-1, mPGES-2, and cPGES were expressed constitutively in neurons; COX-1 was also constitutively expressed in microglia. The immunoreactivity of COX-2 was increased in neurons and astrocytes surrounding blood vessels at 5 h and then tended to decrease in neurons and increase in astrocytes at 1 day. At 3 days after ICH, COX-2 was observed primarily in astrocytes but was absent in neurons. Interestingly, the immunoreactivity of mPGES-1 was increased in neurons in the ipsilateral cortex and astrocytes in the ipsilateral striatum at 1 day post-ICH; the immunoreactivity of astrocytic mPGES-1 further increased at 3 days.

**Conclusion:**

Our data suggest that microglial COX-1, neuronal COX-2, and astrocytic COX-2 and mPGES-1 may work sequentially to affect ICH outcomes. These findings have implications for efforts to develop anti-inflammatory strategies that target COX/PGES pathways to reduce ICH-induced secondary brain damage.

## Background

Intracerebral hemorrhage (ICH) is a subtype of stroke that carries high morbidity and mortality. ICH causes brain injury through primary physical disruption of adjacent tissue and the mass effect and secondary injury such as brain edema and inflammation [1,2]. Evidence from preclinical and clinical studies suggests that inflammatory mechanisms contribute to the progression of secondary brain injury after ICH [1,3]. Inflammatory cells that have the potential to promote hemorrhagic brain damage include blood-derived leukocytes and macrophages, resident microglia, astrocytes, and mast cells. Molecular components of the inflammatory response include prostaglandins, chemokines, cytokines, extracellular proteases, and reactive oxygen species [3-5].

Prostaglandin E_2 _(PGE_2_) is a key component in the initiation and propagation phases of the inflammatory process induced after various types of brain injury [6-8]. It is synthesized from arachidonic acid in two enzymatic steps. Cyclooxygenase (COX) catalyzes the conversion of arachidonic acid to PGH_2_, which is then converted to PGE_2 _by PGE synthase (PGES). COX is present in two isoforms, constitutive COX-1 and inducible COX-2. Under normal conditions, COX-1, which is expressed in numerous tissues, has been classically considered to be the isoform principally responsible for homeostatic prostaglandin synthesis [9]. In contrast, COX-2 is primarily expressed in glutamatergic neurons, wherein it is thought to participate in synaptic signaling [10]. Three major isozymes of PGES have been isolated: microsomal PGES (mPGES)-1, mPGES-2, and cytosolic PGES (cPGES). Of these, mPGES-2 and cPGES are constitutively expressed in various cells and tissues; only mPGES-1 can be induced by pro-inflammatory stimuli and in ischemic stroke models [7,11-13]. A recent study showed that COX-2 and mPGES-1 are co-induced by excess glutamate in ischemic brain and that they act together to exacerbate stroke injury by amplifying PGE_2 _production [14].

Although COX/prostaglandin signaling has been implicated in the pathologic progression of ischemic stroke [15-17], research on their role in ICH is limited. To our knowledge, no reports have been published regarding expression of COX-1 and PGES isoforms in ICH. Two studies have been published on COX-2 expression in blood models of ICH in rats, but the results are conflicting. One showed COX-2 immunoreactivity to be increased from 6 h to 3 days after ICH [18], whereas the second showed a transient increase in COX-2 from 1 to 3 h after ICH followed by a significant down-regulation [19]. To lay a foundation for understanding the roles of COX and PGES isoforms in ICH pathology, here we characterized the expression and cellular localization of COX-1, COX-2, mPGES-1, mPGES-2, and cPGES from 5 h to 3 days after collagenase-induced ICH in mice.

## Materials and methods

### Animals

This study was conducted in accordance with the National Institutes of Health guidelines for the use of experimental animals. Experimental protocols were approved by the Johns Hopkins University Animal Care and Use Committee. A total of 56 C57BL/6 male mice (25-35 g) were obtained from Charles River Laboratories (Wilmington, MA). All efforts were made to minimize the numbers of animals used and ensure minimal suffering.

### ICH model

The procedure for modeling ICH by intrastriatal injection of collagenase was adapted to mice from an established rat protocol [20] and has been described previously [21,22]. Briefly, C57BL/6 mice weighing 22-35 g were anaesthetized by isoflurane (3.0% for induction, 1.0% for maintenance) and ventilated with oxygen-enriched air (20%:80%) via a nose cone. We then injected mice in the left striatum with collagenase VII-S (0.075 U in 500 nL saline, C2399, Sigma, St. Louis, MO) at the following stereotactic coordinates: 1.0 mm anterior and 2.2 mm lateral of the bregma, 2.7 mm in depth. Collagenase was delivered over 5 min. The needle was held in place for an additional 5 min to prevent reflux. Sham-operated mice were injected with saline only. Rectal temperature was monitored and maintained at 37.0 ± 0.5°C with a heating pad throughout the experimental and recovery periods. This procedure resulted in reproducible lesions that were mostly restricted to the striatum.

### Preparation of brain slices

At 5, 24, or 72 h after ICH, 3 mice per group were anesthetized deeply with phenobarbital and perfused transcardially with phosphate-buffered saline (PBS), pH 7.4. Sham-operated control mice (n = 2 at each time point) were perfused similarly. The brains were harvested and fixed with 4% paraformaldehyde overnight, cryoprotected in serial phosphate-buffered sucrose solutions (20%, 30%, and 40%) at 4°C, and then cut into 20-μm sections with a cryostat.

### Hemorrhagic injury volume

Mice (n = 5) were euthanized at 24 h after ICH. The entire brain of each mouse was cut into 50-μm sections with a cryostat. Sections were stained with Luxol fast blue (for myelin) and Cresyl Violet (for neurons) before being quantified for grey and white matter injury with SigmaScan Pro software (version 5.0.0 for Windows; Systat, San Jose, CA). The injury volume in cubic millimeters was calculated by multiplying the thickness by the damaged areas of each section [21].

### Immunofluorescence

Immunofluorescence was carried out as described previously [23,24]. Briefly, free-floating sections were washed in PBS for 20 min, blocked in 5% normal goat serum, and incubated overnight at 4°C with primary antibodies: rabbit anti-COX-1 (1:250; Catalog No. 160109, Cayman Chemical, Ann Arbor, MI); rabbit anti-COX-2 (1:250; Catalog No. 160106, Cayman Chemical); rabbit anti-mPGES-1 (1:250; Catalog No. 160140, Cayman Chemical); rabbit anti-mPGES-2 (1:250; Catalog No. 160145, Cayman Chemical); rabbit anti-cPGES (1:250; Catalog No. 10209, Cayman Chemical); mouse anti-NeuN (1:500; Catalog No. MAB377, Chemicon, Temecula, CA), specific for neurons; rat anti-GFAP (1:250; Catalog No. 13-0300, Invitrogen, Carlsbad, CA), specific for astrocytes; and rat anti-CD11b (1:1000; Catalog No. MCA711G, Serotec, Raleigh, NC), specific for microglia/macrophages. The sections were then washed with PBS and incubated with Alexa-488 (1:1000; Molecular Probes, Eugene, OR)- and/or Cy3 (1:1000; Jackson Labs, West Grove, PA)-conjugated secondary antibodies for 60 min. Stained sections (n = 3/mouse) were examined with a fluorescence microscope; the images were captured from the frontoparietal cortex and striatum and analyzed by SPOT advanced image software (Diagnostic Instruments Inc., Sterling Heights, MI). Control sections were processed identically, except that primary antibodies were omitted. Control sections lacked specific staining. The specificity of the antibodies against COX-1, COX-2, mPGES-1, mPGES-2, and cPGES was further confirmed by using the corresponding blocking peptides (Cayman Chemical).

To quantify the number of immunoreactive cells labeled with COX-1, COX-2, mPGES-1, mPGES-2, and cPGES, three sections per mouse (from the injection site and 360 μm on each side; n = 3 mice/group) were analyzed in the perihematoma region of the striatum. This region was defined within one 20× field that corresponded to ~460 *μ*m from the edge of the hematoma. Positively stained cells were counted in three comparable, randomly selected 30× microscopic fields. The numbers of immunoreactive cells from nine locations per mouse (3 fields per section × 3 sections per mouse) were averaged and expressed as positive cells per field (30×).

### Statistics

All data are expressed as means ± SD. The statistical comparisons among multiple groups were made using one-way ANOVA followed by Bonferroni correction. Statistical significance was set at *P *< 0.05.

## Results

### Brain injury volume

Collagenase injection produced a hematoma that was primarily restricted to the striatum. All mice displayed obvious neurobehavioral deficits within hours of ICH onset (i.e., ipsilateral turning bias). At 24 h after ICH, the brain injury volume calculated from serial sections stained with Luxol fast blue/Cresyl Violet via an image analysis system was 7.5 ± 1.6 mm^3 ^(n = 5).

### Expression of COX-1 and COX-2 after ICH

**Figure 1:**
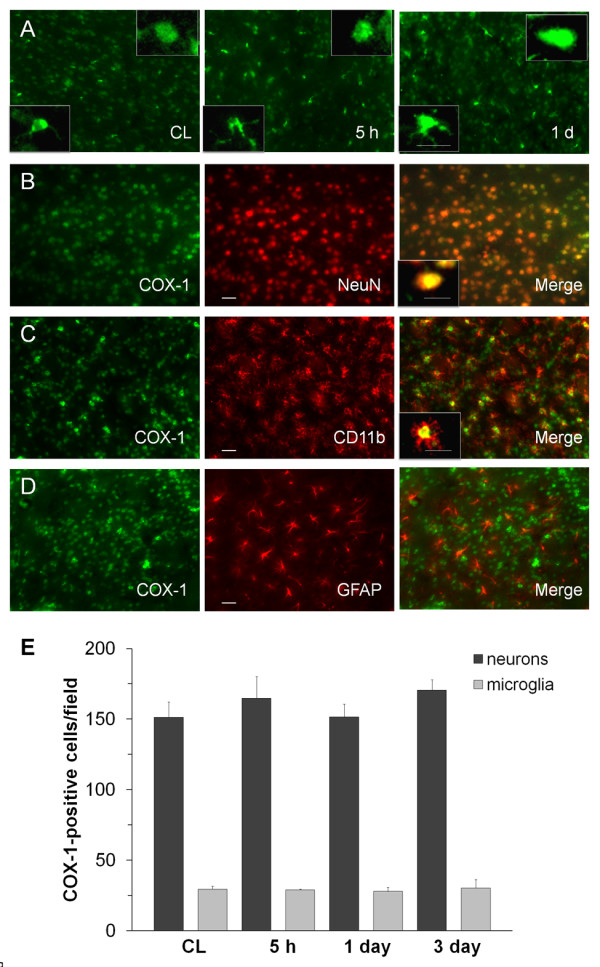
**Immunostaining of COX-1 and cell type-specific proteins in the mouse brain after ICH**. A: COX-1 was constitutively expressed in the contralateral striatum (CL) and did not change significantly from 5 h to 1 day after ICH. The insets in A are representative COX-1-expressing cells at higher magnification (Scale bar, 10 μm). B-D: Double immunostaining of COX-1 with cell type-specific markers for neurons (NeuN), microglia (CD11b), and astrocytes (GFAP) in the striatum surrounding the hematoma at 3 days after ICH. Insets in B and C show a double-stained cell at higher magnification (Scale bar, 10 μm). Scale bar in the middle panels of B, C, D, 30 μm. E: Quantification analysis confirmed that the number of COX-1-immunoreactive neurons and microglia did not change in the perihematomal region of the striatum from 5 h to 3 days after ICH (n = 3/group, all *P *> 0.05). Values are means ± S.D.

COX-1 immunoreactivity was primarily observed in neuron-like and glia-like cells in the contralateral and ipsilateral striatum (figure 1A) and did not change significantly from 5 h to 3 days after ICH (figure 1A-D). To determine the cellular location of COX-1 following ICH, we double stained serial sections obtained 3 days after ICH for COX-1 and cell-specific antigens. The results revealed that COX-1 immunoreactivity was mostly associated with neurons (figure 1B) and microglia (figure 1C). Co-localization was not observed with GFAP, a marker for astrocytes (figure 1D). Cell count analysis confirmed that the number of COX-1-immunoreactive neurons and microglia did not change from 5 h to 3 days in the perihematomal region of the striatum (figure 1E, n = 3/group, all *P *> 0.05).

**Figure 2:**
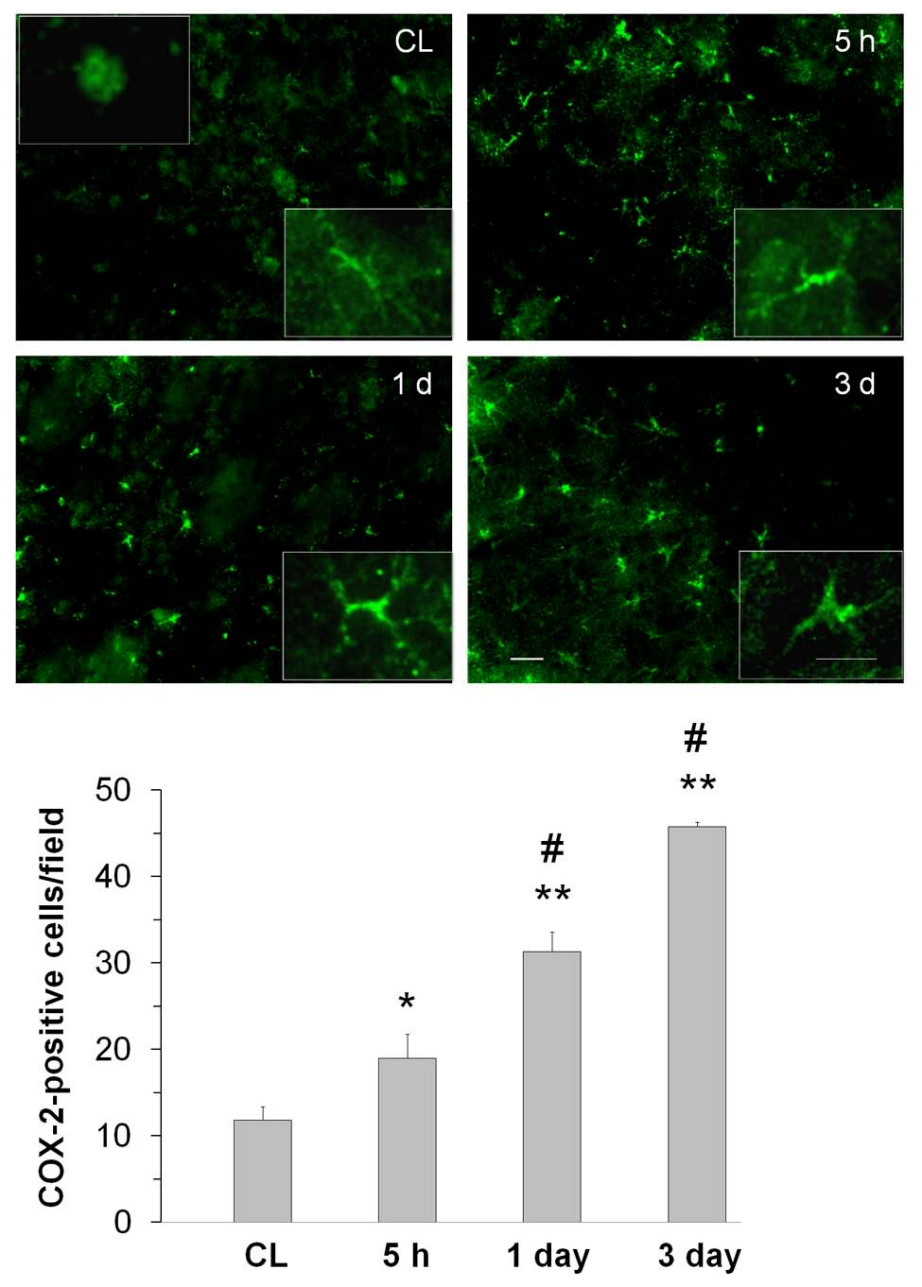
**Immunostaining of COX-2 in the mouse brain after ICH**. Minimal COX-2 immunoreactivity was observed in neuron-like and glia-like cells in the contralateral striatum (CL). After ICH, the number of strongly COX-2-immunoreactive cells increased in the perihematomal region of the striatum; the increase started at 5 h and continued at 3 days. Scale bar, 30 μm. Insets are representative COX-2-expressing cells at higher magnification (Scale bar, 15 μm). Quantification analysis confirmed that the number of strongly COX-2-immunoreactive cells increased from 5 h to 3 days in the perihematomal region of the striatum (n = 3/group, * *P *< 0.05, ** *P *< 0.01 compared with the CL, ^# ^*P *< 0.01 compared with the previous time point). Values are means ± S.D.

**Figure 3:**
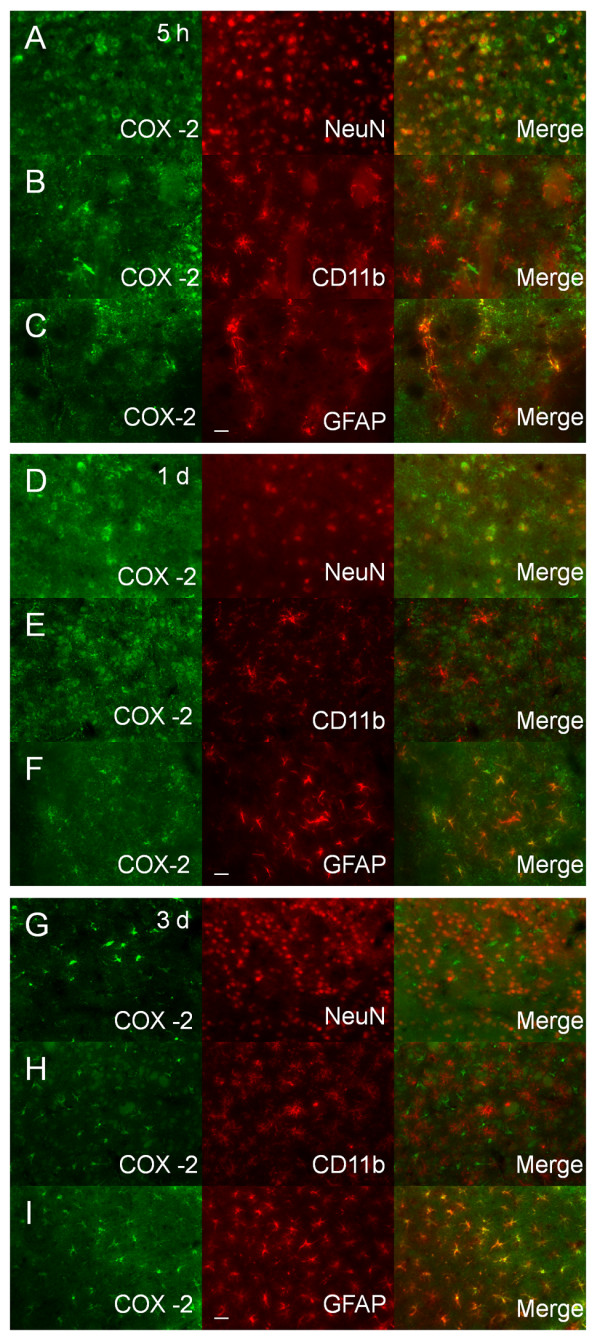
**Double immunostaining of COX-2 with cell type-specific markers (NeuN for neurons, CD11b for microglia, and GFAP for astrocytes) in the mouse brain after ICH**. A-C: In the perihematomal region of the striatum, the number of COX-2-immunoreactive cells increased; double immunostaining demonstrated that COX-2 was expressed in neurons (A) and astrocytes (C), but not in microglia (B), at 5 h after ICH. D-F: At 1 day after ICH, the COX-2 immunoreactivity tended to decrease in neurons (D) and increase in astrocytes (F) of the perihematomal region in the striatum; however COX-2 immunoreactivity remained unchanged in microglia (E). G-I: At 3 days post-ICH, COX-2 immunoreactivity further increased in astrocytes (I) but was not observed in neurons (G) or microglia (H) in the perihematomal region of the striatum. Scale bar in C, F, I, 30 μm.

Immunoreactivity for COX-2 in neuron-like and glia-like cells was minimal in the contralateral striatum (figure 2). After ICH, the number of COX-2-immunoreactive cells increased in the perihematomal region of the striatum; the increase was present at 5 h and continued at 3 days, a finding confirmed by quantification analysis. COX-2-immunoreactive astrocytes displayed increasing hypertrophy of the cell bodies and processes from 5 h to 3 days after ICH (insets in figure 2). In the perihematomal region of the striatum, the immunoreactivity of COX-2 was significantly increased in neurons and astrocytes surrounding blood vessels at 5 h. The intensity tended to decrease in neurons and increase in astrocytes at 1 day after ICH. At 3 days, the immunoreactivity of COX-2 continued to increase in astrocytes but was nearly undetectable in neurons (figure 3). COX-2 immunoreactivity was not detected in microglia at any time point studied (figure 3).

### Expression of mPGES-1, mPGES-2, and cPGES after ICH

**Figure 4:**
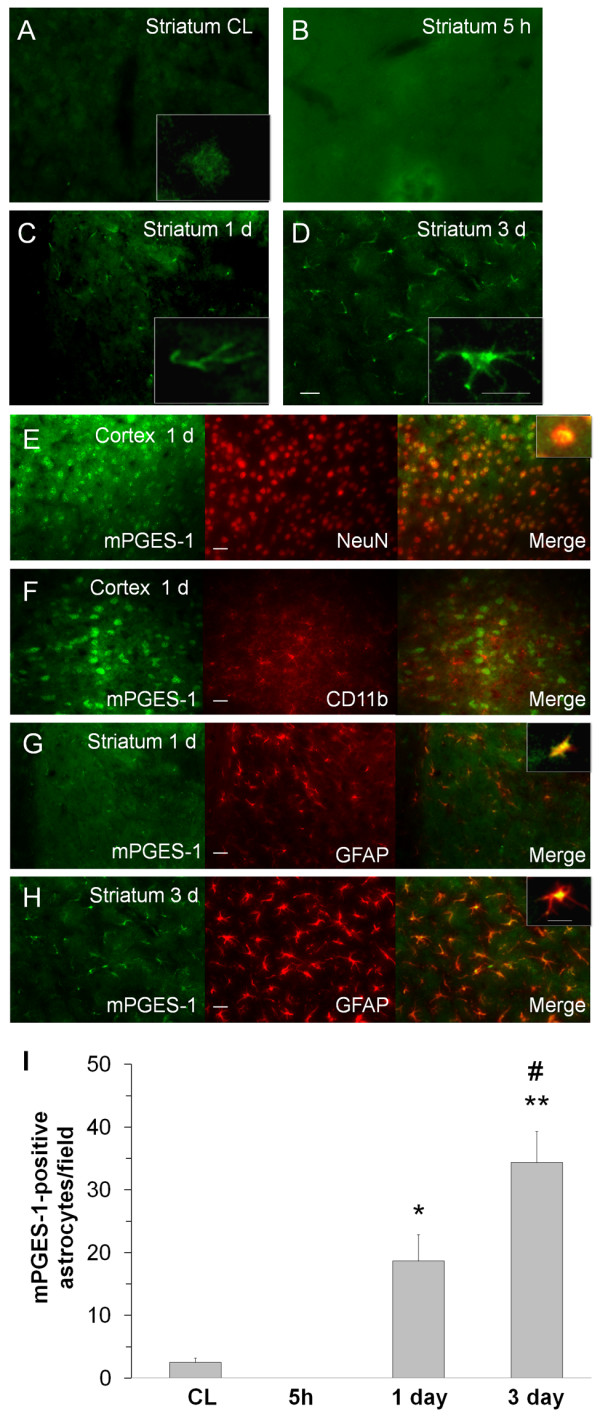
**Immunostaining of mPGES-1 and cell type-specific proteins (NeuN for neurons, CD11b for microglia, and GFAP for astrocytes) in the mouse brain after ICH**. (A) Minimal immunoreactivity of mPGES-1 was detected in neuron-like cells in the contralateral striatum (CL). B-D: In the perihematomal region of the striatum, mPGES-1 immunoreactivity was undetectable at 5 h (B) but began to increase in glia-like cells at 1 day (C). It was additionally increased in glia-like cells at 3 days (D). Insets in A, C, and D are representative mPGES-1-expressing cells at higher magnification (Scale bar, 15 μm). E-F: Double immunostaining showed induction of mPGES-1 in neurons (E), but not in microglia (F), in the ipsilateral frontal-parietal cortex at 1 day. G-H: In the perihematomal region of the striatum, double immunostaining showed that mPGES-1 was induced primarily in astrocytes at 1 day (G); the number of mPGES-1-immunoreactive astrocytes was additionally increased at 3 days (H). Scale bar in D-H, 30 μm. Insets in E, G, and H are double-stained cells at higher magnification (Scale bar, 15 μm). Quantification analysis (I) confirmed that the number of mPGES-1 immunoreactive cells increased at 1 day, and further increased at 3 days in the perihematomal region of the striatum (n = 3/group, * *P *< 0.05, ** *P *< 0.01 compared with the CL, ^# ^*P *< 0.01 compared with day 1). Values are means ± S.D.

Minimal immunoreactivity of mPGES-1 was observed in neuron-like cells in the contralateral cortex (data not shown) and striatum (figure 4A). In the perihematomal region, the immunoreactivity of mPGES-1 began to increase in glia-like cells at 1 day (figure 4C) and was further increased at 3 days after ICH (figure 4D). In the ipsilateral cortex, mPGES-1 immunoreactivity was observed primarily in neurons (figure 4E), infrequently in astrocytes (data not shown), and rarely in microglia/macrophages (figure 4F) at 1 day after ICH. In the ipsilateral striatum, however, mPGES-1 immunoreactivity was detected primarily in astrocytes; it was elevated at 1 day (figure 4G) and further increased at 3 days (figure 4H), as confirmed by quantification analysis (Figure 4I). mPGES-1 was not detected in neurons (data not shown).

**Figure 5:**
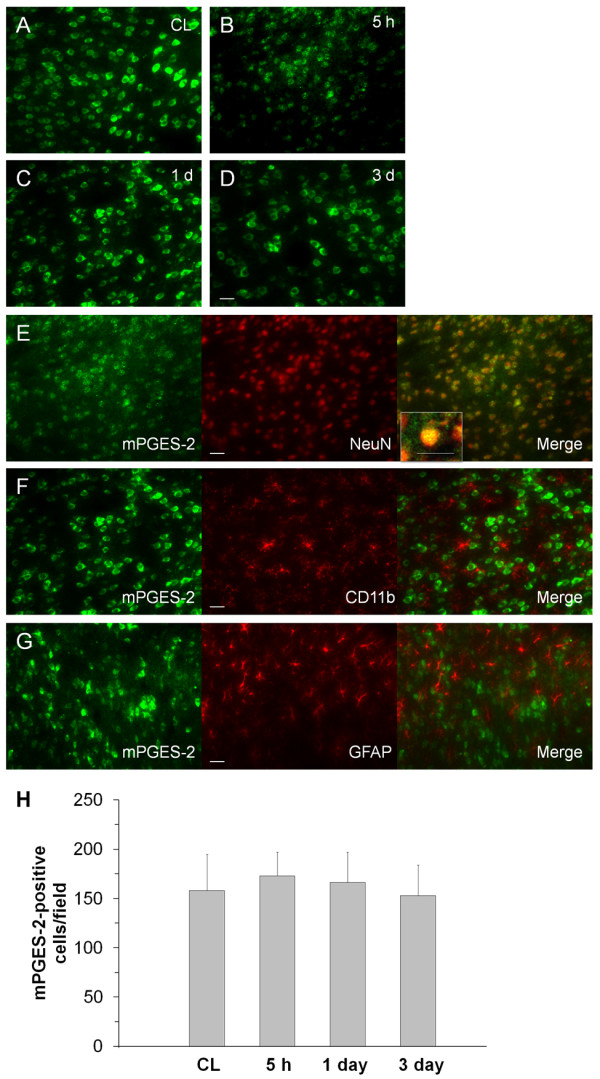
**Immunostaining of mPGES-2 and cell type-specific proteins (NeuN for neurons, CD11b for microglia, and GFAP for astrocytes) in the mouse brain after ICH**. A: Immunoreactive staining for mPGES-2 was observed in neuron-like cells in the contralateral striatum (CL). B-D: In the perihematomal region of the striatum, mPGES-2 immunoreactivity did not change significantly between 5 h (B) and 1 day (C) or 3 days (D) post-ICH. E-G: In the perihematomal region of the striatum, double immunostaining revealed that mPGES-2 was present in neurons at 1 day (E), but not in microglia at 1 day (F) or astrocytes at 3 days (G). Scale bar in D-G, 30 μm. Inset in E is a double-stained cell at higher magnification (Scale bar, 20 μm). Quantification analysis (H) confirmed that the number of mPGES-2-immunoreactive cells did not change in the perihematomal region of the striatum from 5 h to 3 days after ICH (n = 3/group, all *P *> 0.05). Values are means ± S.D.

mPGES-2 was constitutively expressed in neuron-like cells in the contralateral and ipsilateral striatum (figure 5A-D) and did not change significantly between 5 h and 3 days after ICH (figure 5B-D). Double immunofluorescence labeling revealed that the mPGES-2 was located primarily in neurons at 5 h after ICH (figure 5E) but that it was absent from microglia/macrophages at 1 day (figure 5F) and astrocytes at 3 days (figure 5G) after ICH. Cell count analysis confirmed that the number of mPGES-2-immunoreactive cells did not change from 5 h to 3 days in the perihematomal region of the striatum (figure 5H, n = 3/group, all *P *> 0.05).

**Figure 6:**
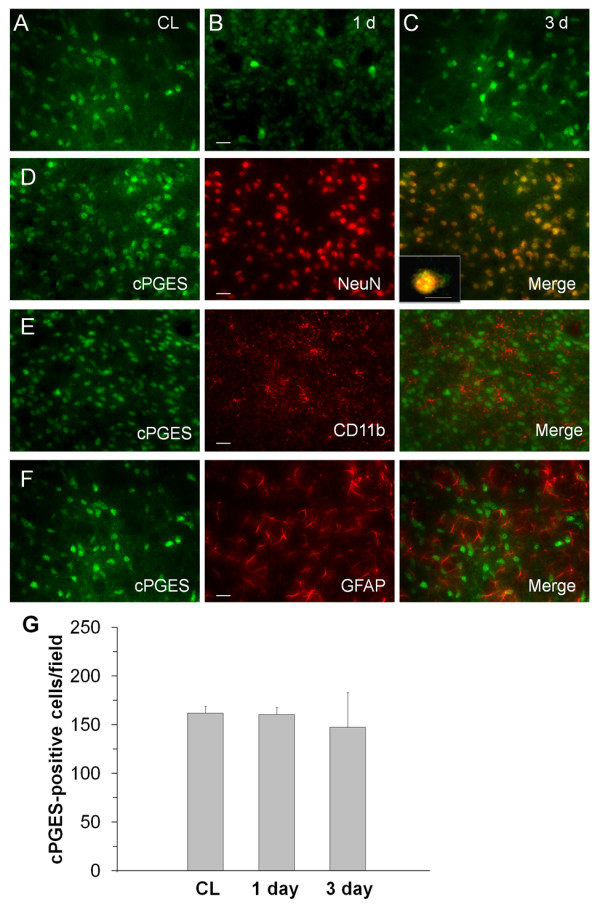
**Immunostaining of cPGES and cell type-specific proteins (NeuN for neurons, CD11b for microglia, and GFAP for astrocytes) in the mouse brain after ICH**. A: Immunoreactive staining for cPGES was observed in neuron-like cells in the contralateral striatum (CL). B-C: In the perihematomal region of the striatum, cPGES immunoreactivity did not change significantly between 1 day (B) and 3 days (C). D-F: In the perihematomal region of the striatum, double immunostaining showed that cPGES was present in neurons (D), but not in microglia (E) or astrocytes (F), at 1 day. Scale bar in B-F, 30 μm. Inset in D is a double-stained cell at higher magnification (Scale bar, 15 μm). Quantification analysis (G) confirmed that the number of cPGES immunoreactive cells did not change in the perihematomal region of the striatum between 1 day and 3 days after ICH (n = 3/group, all *P *> 0.05). Values are means ± S.D.

Similar to mPGES-2, immunostaining revealed cPGES to be expressed constitutively in neuron-like cells in the contralateral and ipsilateral striatum (figure 6A-C); it did not change significantly between 1 and 3 days after ICH (figure 6B-C). Double immunofluorescence labeling indicated that cPGES was present primarily in neurons (figure 6D), but not in microglia/macrophages (figure 6E) or astrocytes (figure 6F) at 1 day after ICH. Cell count analysis confirmed that the number of cPGES-immunoreactive cells did not change from 1 day to 3 days in the perihematomal region of the striatum (figure 6G, n = 3/group, all *P *> 0.05).

## Discussion

To our knowledge, this is the first systematic study performed to characterize the expression and cellular localization of COX and PGES isozymes in the hemorrhagic brain of mice. Using immunofluorescence staining, we observed constitutive expression of COX-1, mPGES-2, and cPGES in neurons; COX-1 was also constitutively expressed in microglia. In contrast, COX-2 and mPGES-1 immunoreactivity, which was minimal in the normal brain, underwent distinct time-dependent changes in neurons and astrocytes of the perihematomal region during the first 3 days post-ICH. Our data support the premise that the COX/PGES signaling pathway contributes to ICH pathology.

COX-1 is constitutively expressed in most tissues [9]. We demonstrated for the first time that COX-1 is constitutively expressed in neurons and microglia in the hemorrhagic brain. Although involvement of COX-1 in ICH pathology has not been studied, the evidence that COX-1 is produced in microglia of the perihematomal region implies a toxic role of COX-1 in the pathophysiology of the disease. This hypothesis is based on the fact that activated microglia/macrophages are the major sources of proinflammatory mediators [1,3] and that inhibition of microglial activation before or early after ICH decreases neuronal death and improves neurologic function [21,25]. We therefore propose that microglial COX-1 might immediately initiate synthesis of prostaglandins in response to microglial activation and could be considered one of the major players in mediating neuroinflammation after ICH.

In the normal brain, COX-2 is constitutively expressed in neurons of the cortex, hippocampus, and striatum [8]. COX-2 is mainly induced in response to inflammatory stimuli; deletion of the COX-2 gene or selective COX-2 inhibition reduces infarction volume and neuronal death after cerebral ischemia [26-28]. To our knowledge, only two studies have investigated post-ICH COX-2 expression, and the results were conflicting [18,19]. Zhao et al. [19] demonstrated that COX-2 mRNA and protein were increased within 3 h after ICH and that COX-2 immunoreactivity was increased in blood vessels and neurons in the perihematomal region at 4 h; the increase in immunoreactivity was transient, followed by a significant down-regulation at days 1 and 3. In the present study, we found that the immunoreactivity of COX-2 in astrocytes increased gradually in the perihematomal region from 5 h to 3 days, whereas neuronal COX-2 increased only transiently at 5 h after ICH. Most of our results are consistent with the findings by Gong et al. [18], except that we found COX-2 to be increasingly induced in astrocytes from 1 to 3 days, whereas Gong et al. reported that COX-2 was induced in endothelial cells, perivascular cells, and infiltrating leukocytes at 1 day after blood infusion. The reason for the discrepancy between the previous studies and our own is not clear, but it may be a result of differences in ICH models and species used and the size of the intrastriatal hematoma formed. In the two previous studies, investigators modeled ICH by injecting rats intrastriatally with differing amounts of autologous blood. In contrast, we used a collagenase-induced ICH model in mice that may cause gradual hematoma growth over the first few hours with subsequent inflammation. Our data suggest that astrocytic COX-2 in concert with microglial COX-1 might contribute to collagenase-induced post-hemorrhagic neuroinflammation. More studies are warranted to understand whether the functions of neuronal and astrocytic COX-2 differ after ICH.

Induction of mPGES-1 expression has been observed in various conditions, such as inflammation, fever, pain, tissue repair, and cancer, in which COX-2-derived PGE_2 _plays a critical role [29]. In the ischemic brain, mPGES-1 and COX-2 are both induced in neurons, microglia, and endothelial cells in the ipsilateral cerebral cortex and striatum [7]. It has been confirmed that mPGES-1 and COX-2 are co-localized and co-induced in the infarct region of the cortex, and it has been suggested that they act together to exacerbate stroke injury [14]. At 1 day post-ICH in our model, mPGES-1 expression in the ipsilateral cortex was elevated primarily in neurons whereas in the perihematomal region, it was elevated in astrocytes. Astrocytic expression continued to increase for at least 3 days. However, we observed no apparent changes in the expression of neuronal mPGES-2 or cPGES. The different cellular expression profiles for mPGES-1 between cortex and striatum suggests that mPGES-1 may be involved in different signaling pathways within the cortical neurons and striatal astrocytes after ICH. In line with the results from a lipopolysaccharide-induced inflammation model [30], we found that the induction of COX-2 protein expression was more rapid than that of mPGES-1 in the hemorrhagic brain. These results suggest that the sequential up-regulation and co-induction of COX-2 and mPGES-1 in astrocytes in the perihematomal region might contribute to inflammation-mediated secondary brain injury after ICH, possibly through excessive PGE_2 _production.

In conclusion, our data provide novel evidence that COX-1, mPGES-2, and cPGES are constitutively expressed in the hemorrhagic brain; COX-2 is induced early in neurons and later in astrocytes. Although neuronal COX-2 is induced earlier than astrocytic COX-2, the latter is induced in parallel with astrocytic mPGES-1. Together, our data suggest that microglial COX-1, neuronal COX-2, astrocytic COX-2, and astrocytic mPGES-1 may work sequentially to affect ICH outcomes. Based on our previous observations that neuroinflammation affects the normal function of the entire brain in patients with lethal ICH [31], these findings have implications for efforts to develop anti-inflammatory strategies that target the COX/PGES pathway to reduce ICH-induced secondary brain damage. Indeed, a recent study showed that inhibition of COX-2 attenuated inflammation, neuronal death, and gliosis and promoted long-term recovery in motor function and myelination in rabbit pups with intraventricular hemorrhage [32].

## Competing interests

The authors declare that they have no competing interests.

## Authors' contributions

TW, HW, and Jessica W carried out the ICH model and immunofluorescence staining and helped draft the manuscript. Jian W conceived of the study, participated in its design and conduct, and helped draft the manuscript. All authors read and approved the final manuscript.
